# Blunt traumatic aortic injury to the brachiocephalic and left carotid arteries

**DOI:** 10.1186/s44215-022-00013-2

**Published:** 2022-10-17

**Authors:** Yu Inaba, Yasunori Iida, Hidetoshi Oka, Takahisa Miki, Takashi Hachiya, Hideyuki Shimizu

**Affiliations:** 1 Department of Cardiovascular Surgery, Saiseikai Yokohamashi Tobu Hospital, 3-6-1 Shimosueyoshi tsurumiku, Yokohama City, Kanagawa 230-8765 Japan; 2 Department of Cardiovascular Surgery, Keio University, Tokyo, Japan

**Keywords:** Blunt traumatic aortic injury, Brachiocephalic artery, Carotid artery

## Abstract

**Background:**

Blunt traumatic aortic injury (BTAI) is a life-threatening pathology that most commonly occurs after traffic accidents or a fall from a considerable height. We report a rare case of traumatic double transection which included the brachiocephalic artery (BCA) and left carotid artery (LCA) following a motorcycle accident.

**Case presentation:**

A 52-year-old man was brought to our emergency room for blunt trauma. Urgent computed tomography (CT) indicated BCA and LCA transection. Two weeks after the BTAI, CT indicated an increase in the size of the BCA pseudoaneurysm, a newly developed LCA pseudoaneurysm, and a spread of hematoma. An emergency partial aortic arch replacement including BCA and LCA reconstruction was performed 17 days after the BTAI. The postoperative course was good, and he was discharged 12 days later.

**Conclusions:**

An open aortic repair appeared to be more suited in the present case because the aortic injury occurred in a branched lesion of the aortic arch. It was thought that complete stent graft coverage of the transection lesion would be difficult. Although coexisting multiple system injuries complicate aortic repair, there was a rapid enlargement of the BCA and LCA pseudoaneurysms. Therefore, strict management is needed even in the case of cervical artery injury.

## Background

Blunt traumatic aortic injury (BTAI) is a life-threatening pathology that most commonly occurs after traffic accidents or a fall from a considerable height [1]. The most common site of injury is the aortic isthmus distal to the left subclavian artery. Traumatic injury to the cervical artery is rare and caused by penetrating trauma in the majority of cases, although 35% of cases result from blunt trauma [2]. Herein, we report a rare case of traumatic double transection which included the brachiocephalic artery (BCA) and left carotid artery (LCA) following a motorcycle accident.

## Case presentation

**Fig. 1:**
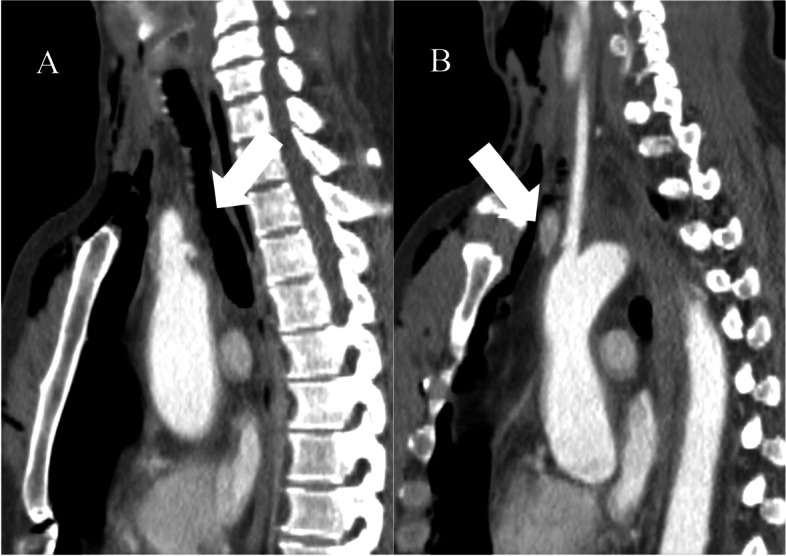
Contrast computed tomographic (CT) images during the emergency transportation. CT showed transections in the brachiocephalic artery (BCA) (**A**) and left carotid artery (LCA) (**B**)

**Fig. 2:**
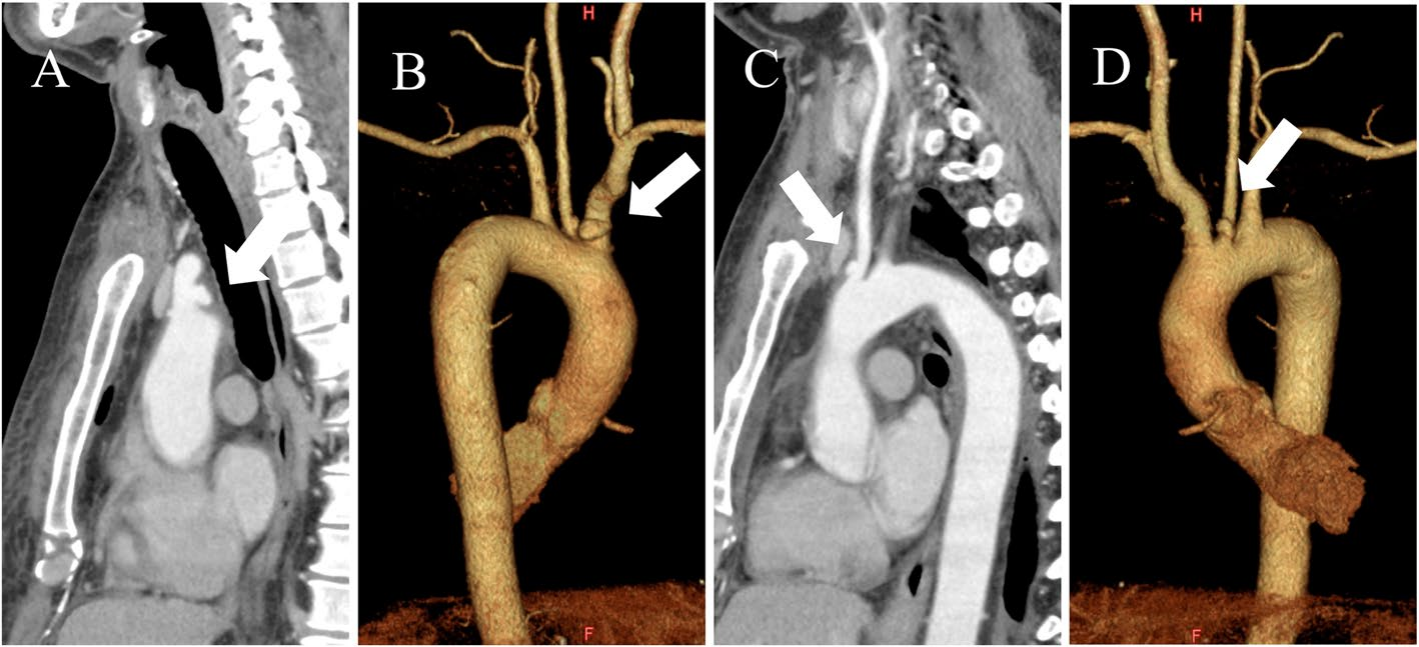
CT scans 2 weeks later following emergency admission. An enlargement of the BCA pseudoaneurysm (white arrows) was detected in a sagittal slice of CT (**A**) and a three-dimension CT (**B**). A newly developed LCA pseudoaneurysm (white arrows) was detected in a sagittal slice of CT (**C**) and a three-dimension CT (**D**)

**Fig. 3:**
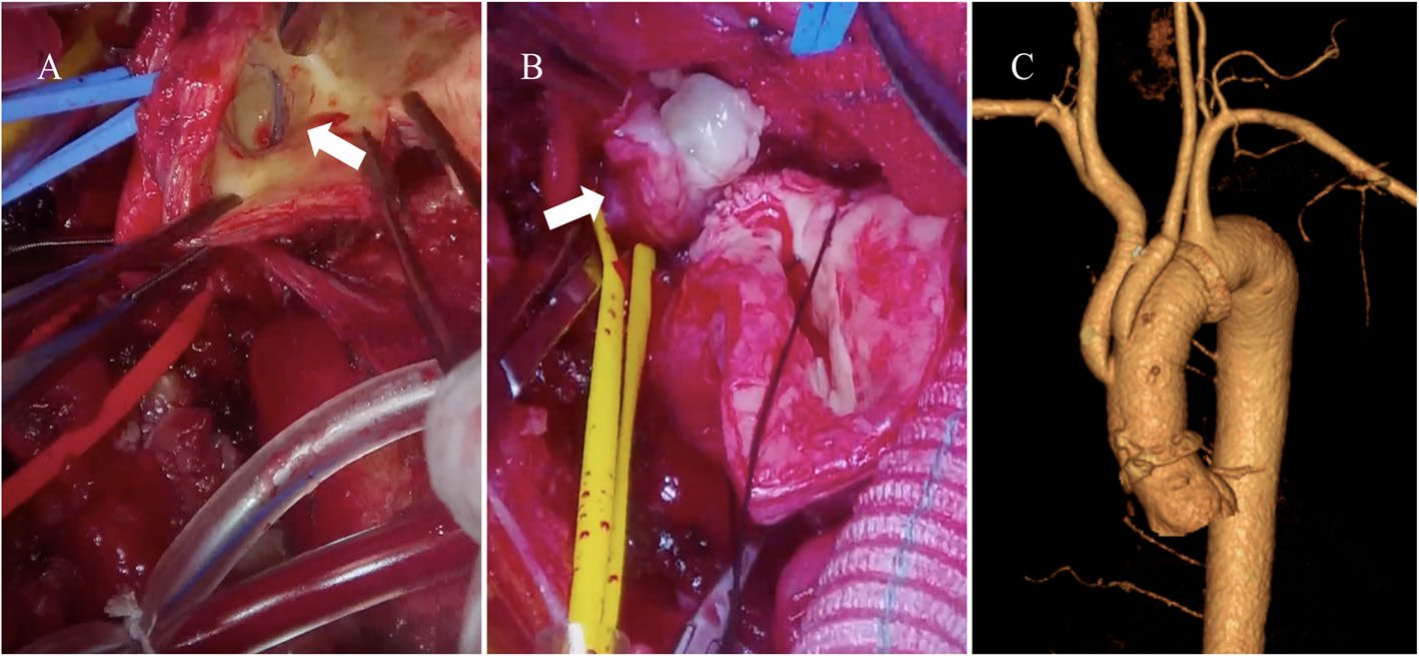
Partial aortic arch replacement was performed 17 days after emergency admission. Surgical view of cervical arteries under cardio-pulmonary bypass. A white arrow indicates branched lesion of the BCA. A half-round intima of the BCA was transected (**A**). The LCA was taped by a yellow tape. A thick hematoma was found around the LCA (**B**). A branched Dacron graft was anastomosed end-to-end to the aorta and cervical vessels (**C**)

A 52-year-old man was brought to our emergency room for blunt trauma. He was riding his motorcycle when he collided head-on with a car at high speed. Urgent computed tomography (CT) indicated BCA and LCA transection (Fig. 1A, B), right clavicle dislocation, bilateral hemothorax, left radial and ulnar fracture, and carotid-cavernous fistula (CCF). Head CT showed no indication of intracranial hemorrhage. He was intubated and chest drainage tubes were inserted bilaterally. One week later, surgical fixation of the radial and ulnar bones was performed. Two weeks after the BTAI, CT indicated an increase in the size of the BCA pseudoaneurysm (Fig. 2A, B), a newly developed LCA pseudoaneurysm, and a spread of hematoma (Fig. 2C, D). An emergency partial aortic arch replacement under deep hypothermia was performed 17 days after the BTAI. Adherent tissue and hematoma were evident in the upper mediastinum, around the aortic arch, and in cervical vessels. A half-round intima of the BCA and LCA was transected (Fig. 3A). A thick hematoma was found around the LCA (Fig. 3B). A branched Dacron graft (J Graft Shield, Japan Lifeline Co., Ltd., Tokyo, Japan) was anastomosed end-to-end to the aorta and cervical vessels (Fig. 3C). The postoperative course was good, and he was discharged 12 days later. Transvenous embolization for CCF was performed 2 weeks later.

## Discussion and conclusion

Traditional views have held that sudden deceleration causes a tear usually near the aortic isthmus [1]. BTAI simultaneously involving the BCA and LCA is extremely rare. Bito and colleagues have reported the incidence of blunt BCA injury was 0 to 2.2% among blunt trauma victims [3]. A proposed mechanism is compression of the mediastinum between the sternum and the vertebrae, resulting in a left-sided displacement of the heart. Concomitant left lateral head rotation and neck hyperextension results in tension in the BCA and LCA [4].

A classification for grading the severity of the aortic injury has been proposed: grade 1 (intimal tear), grade 2 (intramural hematoma), grade 3 (pseudoaneurysm), and grade 4 (rupture) [5]. This case indicated that the BCA pseudoaneurysm had been slightly enlarged and the LCA pseudoaneurysm had been newly developed for 2 weeks of follow-up. So, the present case is thought to be an unstable BTAI grade 3. The Society for Vascular Surgery recommends urgent repair or after other injuries have been stabilized for traumatic aortic injuries [5]. Some have reported that nonoperative management of grade 1 and grade 2 did not result in long-term aortic complications or the need for reintervention. Even the select grade 3 in injury, such as small pseudoaneurysm, minimal hematoma, or those in difficult to treat locations, can be safely followed up with surveillance [6]. Initially, it was thought that this cervical artery injury would not progress similarly to aortic arch injury because these injuries were not the aorta itself and there were small aneurysms. However, there was a rapid enlargement of the BCA and LCA pseudoaneurysms and hematoma. The aortic repair should be performed urgently although coexisting multiple system injuries complicate aortic repair. Therefore, strict management is needed even in the case of cervical artery injury.

In recent years, endovascular aortic repair has become the standard management for BTAI. Endovascular repair is associated with a significantly lower mortality and fewer blood transfusions than open repair [7]. In thoracic traumatic cases, the majorities cases were performed TEVAR without heparinization due to a high risk for bleeding [8]. Moreover, a successful endovascular repair using a kissing technique for traumatic BCA injury has been reported [9]. However, recent meta-analysis found that postoperative mortality was not significantly different between TEVAR and open surgery [10]. Evans and colleagues have reported by a retrospective cohort study of BTAI 427 cases that TEVAR was not associated with improved survival to maximum follow-up when compared with either medical management, hybrid repair, or surgical repair [11]. In the present case, the aortic injury occurred in a branched lesion of the aortic arch. It was thought that complete stent graft coverage of the transection lesion would be difficult. This case favored open surgery under systemic heparinization owing to its anatomical complexity.

## Data Availability

Please contact the author for data request.
